# Real world outcomes in patients with neuroendocrine tumor receiving peptide receptor radionucleotide therapy

**DOI:** 10.37349/etat.2023.00141

**Published:** 2023-06-29

**Authors:** Stijn Hentzen, Kathan Mehta, Raed Moh’d Taiseer Al-Rajabi, Anwaar Saeed, Joaquina Celebre Baranda, Stephen K. Williamson, Weijing Sun, Anup Kasi

**Affiliations:** Istituto Nazionale Tumori-IRCCS-Fondazione G. Pascale, Italy; ^1^Department of Internal Medicine, University of Kansas Medical Center, Kansas City, KS 66160, USA; ^2^Department of Oncology, University of Kansas Cancer Center, Kansas City, KS 66160, USA

**Keywords:** Neuroendocrine tumor, peptide receptor radionucleotide therapy, gastrointestinal cancer, ^177^Lu-dotatate

## Abstract

**Aim:**

^177^Lu-Dotatate (Lu-177), a form of peptide receptor radionuclide therapy (PRRT), was approved by Food and Drug Administration (FDA) for the treatment of somatostatin-receptor-positive neuroendocrine tumors (NETs) in 2018. Clinical trials prior to the FDA approval of Lu-177 showed favorable outcomes but there is limited published real world outcomes data. This study aims to describe and analyze real world outcomes of patients with NET who received Lu-177.

**Methods:**

After obtaining institutional review board approval, retrospective evaluation was performed to analyze the efficacy of Lu-177 for somatostatin receptor-positive gastro-entero-pancreatic NETs (GEP-NETs) patients at the University of Kansas Cancer Center between June 2018 and September 2021. This study aims to determine the response rate to the treatment of the entire cohort and subgroups.

**Results:**

A total of 65 patients received Lu-177 of which 58 completed treatment. The 58 patients had a median age of 61.5 years, 24 females and 34 males, 86% Caucasian and 12% black. The origins of NETs were primarily small bowel (*n* = 24) and pancreatic (*n* = 14). Pathology showed grades 1 (*n *= 21), 2 (*n *= 25), and 3 (*n *= 4) and were primarily well-differentiated tumors (*n *= 4). Among the cohort, 52 patients had imaging to assess response with 14 (26.9%) patients with partial response (PR), 31 (59.6%) with stable disease (SD), and 7 (13.5%) with progressive disease (PD). In a subset analysis, patients with non-functional disease (*n* = 29) had higher rates of PR 42.3% (compared to 11.5%, *P* = 0.0147) and higher disease control rate of 96% (compared to 78%, *P* = 0.042) than patients with functional disease (*n* = 29). Patients with non-functional disease had a lower PD of 3.85% (compared to 23%, *P* = 0.0147) than those with functional disease.

**Conclusions:**

This real world outcomes analysis of NETs treated with Lu-177 shows improved PR when compared to the initial clinical trials and is promising for patients. In addition, patients with non-functional tumors were found to have a statistically significant improved response rate which has not been described in the literature before. If these study findings are validated in a larger cohort they may guide patient selection for Lu-177 therapy in the future.

## Introduction

Gastro-entero-pancreatic neuroendocrine tumors (GEP-NETs) are a prevalent gastrointestinal cancer with increasing incidence [1–3]. Although survival can vary greatly depending on patient- and disease-specific factors, GEP-NETs are associated with significant morbidity and mortality [3]. Treatment with somatostatin analogs remains the standard first-line therapy for somatostatin receptor-positive GEP-NETs due to their highly effective symptom and tumor growth control [4–6]. However, for patients with progressive disease (PD) despite somatostatin analog therapy, treatment options have been limited and lack evidence showing improvement in survival [7]. Fortunately, dramatic advances in the treatment of progressive GEP-NETs have occurred in recent years with the development of peptide receptor radionucleotide therapy (PRRT).

Through the use of radiolabeled somatostatin analogs, PRRT allows targeted radiation to somatostatin receptor-positive tumors [8]. One particular form of this therapy, ^177^Lu-dotatate (Lu-177), was approved by the Food and Drug Administration (FDA) in 2018 [9]. The approval of the therapy was largely based on the NETTER-1 trial which showed longer progression-free survival (PFS) and higher response rate in those treated with Lu-177 compared to high-dose octreotide [10]. In addition to this landmark trial, other clinical trials have shown favorable outcomes with the therapy [11, 12]. These early trials show that the use of PRRT appears promising. An updated analysis of the NETTER-1 cohort was published in 2021. Although the data showed an 11-month increase in median overall survival (OS), the increase was not significant.

Despite FDA approval of Lu-177 in 2018, extensive literature review reveals a lack of publications of real world outcomes. At the University of Kansas Cancer Center, patients with somatostatin receptor-positive GEP-NETs began treatment with PRRT shortly after the approval of Lu-177. These patients have been closely followed by the institution which now has seven oncologists participating in the use of Lu-177. Due to the lack of published real world data, we evaluated all patients who received Lu-177 at our institution. We aimed to provide a descriptive analysis of these patients and evaluate the efficacy of the therapy. In addition to evaluating overall efficacy, we aimed to analyze subgroups to determine whether certain attributes contributed to improved outcomes.

## Materials and methods

### Search strategy

This retrospective study evaluated the outcomes of subjects with somatostatin receptor-positive neuroendocrine tumors (NETs) who were treated with Lu-177 at a tertiary care academic medical center between the dates of June 2018 and September 2021. Prior to data collection, IRB approval was obtained under the ethical IRB number STUDY00147410. Data was collected by a member of the research team, de-identified, and kept secure on a password-protected hard drive. Data was then reviewed by a second member of the team to confirm accuracy.

### Lu-177 administration and response assessment

Per institutional standards, the patients received Lu-177 7.4 GBq (200 mCi) infused by nuclear medicine, over 30–40 min, every 8 weeks for a total of 4 doses. Amino acid solution with arginine and lysine in 1,000 mL was started 30 min before Lu-177 infusion, continued during, and for 3 h after. Anti-emetics were given 30 min prior to the amino acid solution. While receiving Lu-177 patients additionally received long-acting somatostatin analog every 4 weeks. This was administered 4–24 h after each Lu-177 infusion as well as 4 weeks prior to the next Lu-177 dose. Patients were allowed to receive short-acting octreotide in the interim as needed for symptoms, however, this was held within 24 h prior to Lu-177.

Institutional standards for assessment of response to therapy were with computed tomography (CT) imaging 9–12 months from the first dose of Lu-177. The response rate was determined using guidelines outlined by the Response Evaluation Criteria in Solid Tumors (RECIST) [13]. In instances in which patients did not receive the institutional standard CT imaging, gallium-68 dotatate-positron emission tomography (^68^Ga-PET) or magnetic resonance imaging (MRI) or imaging at earlier or later dates were used to determine response.

### Inclusion and exclusion criteria

Patients included those with somatostatin receptor-positive NETs as determined by ^68^Ga-PET scan. Current clinical indication for Lu-177 includes patients with well-differentiated grade 1 or grade 2 GEP-NETs. However, this patient cohort includes patients with grade 3, moderate differentiated, poorly differentiated, and tumors of alternative primary sites due to the decision to treat patients with Lu-177 “off-label” based on ^68^Ga-PET scan positivity. Exclusion criteria included patients who received fewer than four treatments of Lu-177.

### Primary and secondary outcomes

The primary outcome of the study was the response rate defined as partial response (PR), stable disease (SD), or PD. These classifications were determined using RECIST criteria using imaging modalities described above. Statistical analysis was performed using Statistical Analysis System (SAS) technology. *P* values were calculated using the unpaired *t*-test for continuous variables and chi-square test with Yate’s correction for categorical variables. Endpoints of PFS and OS were attempted but unable to be calculated due to the limited duration of follow-up. Final analysis of PFS and OS of this patient cohort will be conducted in the future.

Secondary outcomes included response rate comparison between patients with liver metastases *vs*. those without, patients with functional tumors *vs*. non-functional tumors, patients with pancreas origin *vs*. small bowel origin, and patients with grade 1 *vs*. grade 2 tumors classified using mitotic count and Ki-67 index [14]. The classification of functional tumors *vs*. non-functional tumors was determined by clinical presentation as documented by the patients’ primary oncologist.

## Results

### Descriptive analysis

A total of 65 patients received Lu-177 of which 58 completed treatment and 7 continue to undergo therapy or have yet to receive restaging imaging. Characteristics of this group are shown in Table 1. The 58 patients who completed treatment had a median age of 61.5 years and were composed of 24 females and 34 males. Additionally, the patient population consisted of 86% Caucasian and 12% black patients. These patients primarily had NETs of small bowel (*n* = 24) and pancreatic (*n* = 14) origin with 84% (*n* = 49) of them involving liver metastasis. Based on clinical presentation we found that 50% (*n* = 29) of the patients had functional tumors. Pathology showed 21 patients with grade 1, 25 patients with grade 2, and 4 patients with grade 3 tumors. A large majority of the patients had well-differentiated tumors (*n* = 47).

**Table 1:** Patient characteristics

**Patient characteristic**	**Outcome**
Sex [*n* (%)]	
Male	34 (59)
Female	24 (41)
Age at diagnosis (year)	60
Race [*n* (%)]	
Caucasian	50 (86)
Black	7 (12)
Primary tumor site [*n* (%)]	
Appendix	2 (3)
Colon	3 (5)
Lung	1 (2)
Pancreas	14 (24)
Rectosigmoid	1 (2)
Rectum	2 (3)
Small bowel	24 (41)
Unknown site	11 (19)
Liver metastasis [*n* (%)]	
Present	49 (84)
Absent	9 (16)
Grade* [*n* (%)]	
1	21 (38)
2	25 (45)
3	4 (7)
Differentiation** [*n* (%)]	
Well	47 (90)
Moderate	2 (4)
Poor	1 (2)
Eastern Cooperative Oncology Group (ECOG) score at first treatment	0.58
Prior treatment [*n*]	
Octreotide	47
Lanreotide	3
Everolimus	8
Denosumab	3

*: tumor gradings are defined as 1 = mitotic rate < 2 mitosis/2 mm^2^ and Ki67 index < 3%; 2 = mitotic rate 2–20 mitosis/2 mm^2^ and Ki67 index 3–20%; 3 = mitotic rate > 20 mitosis/2 mm^2^ and Ki67 index > 20% [14]; **: well-differentiated tumors demonstrated low mitotic rate and low-grade bland histology. Moderate-differentiated tumors demonstrated moderate mitotic rate and moderate-grade histology. Poorly differentiated neuroendocrine carcinoma demonstrated a high-mitotic rate and high-grade histology similar in appearance to small cell carcinomas [15]

In addition, data was gathered regarding the treatments prior to and after Lu-177. A large majority of the patients (*n* = 47) received octreotide prior to Lu-177. Three of the patients received lanreotide rather than octreotide. Seven of the patients received everolimus. After Lu-177, 4 of the patients received folinic acid, fluorouracil, and oxaliplatin (FOLFOX) and 3 of the patients received 2 further doses of Lu-177.

### Response analysis

**Figure 1:**
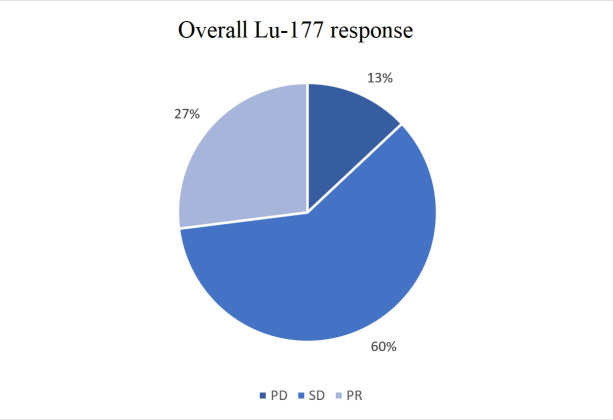
Overall Lu-177 response

In the entire cohort, response assessment was determined at the first imaging studies obtained after four completed treatments with Lu-177 or the last completed treatment of therapy. Of the 58 patients who completed therapy, 52 received restaging imaging. Six patients did not receive restaging imaging due to loss of follow-up (*n* = 4) and death (*n* = 2). Among those who received response assessment, imaging was performed on average 2.1 months from the last treatment with a range of from 0.5 months to 7 months. Imaging modalities used for response assessment included CT (*n* = 26, 50%), positron emission tomography (PET)/CT (*n* = 20, 38%), and MRI (*n* = 6, 12%). We found that 14 (26.9%) of the patients had PR, 31 (59.6%) had SD, and 7 (13.5%) had PD (Figure 1). We reviewed long-term follow-up of the patient cohort extending over three years and found that 40 (69%) of the patients had either PR or SD on their most recent imaging reports. At the final time of data collection, 15 of the patients had died. At the time of analysis, median PFS and OS were not reached.

#### Subgroup response analysis

**Figure 2:**
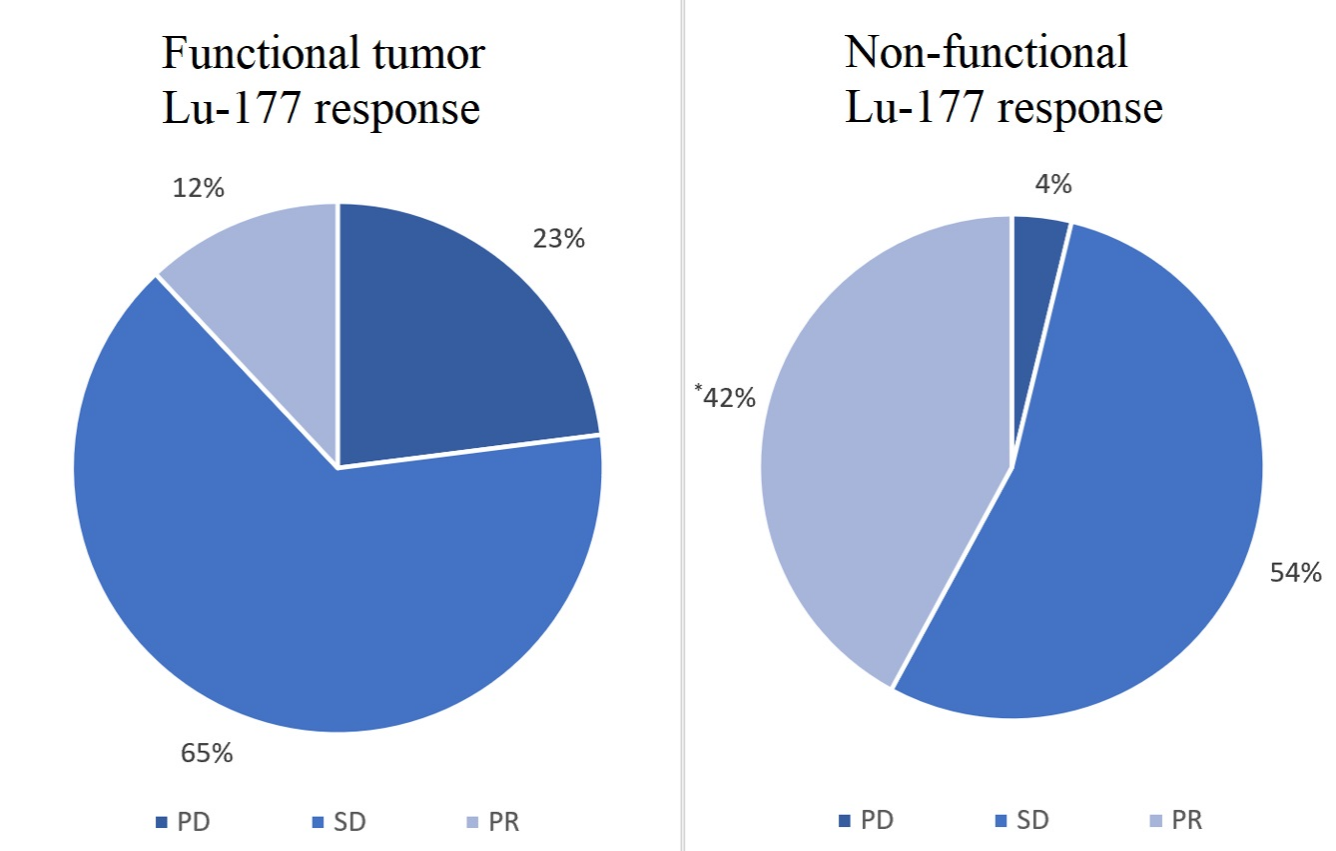
Comparison of functional tumor *vs.* non-functional tumor Lu-177 response. ^*^ Significantly higher rate of PR in patients with non-functional tumors compared to those with functional tumors (*P* = 0.0147)

We also conducted a subset analysis to evaluate the secondary outcomes outlined above. We found no difference in response between patients with and without liver metastases (*P* = 0.5734), no difference between patients with grade 1 *vs*. grade 2 tumors (*P* = 0.7630), and no difference between patients with origins of pancreas *vs*. small bowel (*P* = 0.2604). However, patients with non-functional disease had a significantly higher rate of PR (42.3%) compared to those with functional disease (11.5%, *P* = 0.0147, Figure 2). Additionally, patients with non-functional disease had a higher disease control rate of 96% *vs*. 78% (*P* = 0.042) than those with functional disease. Patients with non-functional disease had a lower PD rate than those with functional disease at 3.85% *vs*. 23% (*P* = 0.0147). Clinical characteristics of these patients are shown in Table 2. Aside from the functional status of the tumor, no statistically significant differences were found among the characteristics of the two groups.

**Table 2:** Comparison of patient characteristics with functional *vs.* non-functional tumors

**Characteristic**	**Functional**	**Non-functional**	***P* value**
Number of patients	29	29	-
Mean age (years)	60	59	0.8
Male (%)	62%	55%	0.8
Small bowel primary	14 (48%)	10 (34%)	0.4
Pancreatic primary	4 (24%)	10 (34%)	0.1
Liver metastasis present	22 (76%)	26 (90%)	0.3
Grade 1	13 (45%)	9 (31%)	0.4
Grade 2	8 (28%)	16 (55%)	0.1
Prior somatostatin analogue treatment	27 (93%)	22 (76%)	0.1
Prior chemotherapy treatment	5 (17%)	12 (41%)	0.1

-: not applicable

### Tumors with unknown primary

A total of 11 patients with NET of an unknown primary received Lu-177 among this cohort. These patients had an average age of 54 years (range 35–80 years). Nine of the patients had well-differentiated tumors while one had poorly differentiated tumor and one patient had undefined differentiation. In response to Lu-177, follow-up imaging showed 1 (9%) patient with PR, 6 (55%) with SD, 3 (27%) with PD, and 1 (9%) who was lost to follow-up. At the time of data collection, 3 (27%) of these patients had died an average of 6.5 months from the first cycle of Lu-177.

## Discussion

The results of this study demonstrate favorable response rates for patients with GEP-NETs treated with Lu-177. A large majority of the patients had either PR or SD at the first imaging study after four treatments. Due to the extended follow-up of these patients, we were also able to analyze the long-term effects of the therapy. We found that over a follow-up period greater than three years, these patients continued to have PR or SD. Given that trial population fitness is often far superior to the fitness of patients in the real world these findings validate the results of NETTER-1 and other early randomized controlled trials (RCTs) which supported the use of PRRT [16].

In a closer review of the results, we find that the response rate of the patient cohort described here is even superior to the findings of the early RCTs [10]. The patients included in this study had a PR rate of 26.9% in comparison to a response rate of 18% in the NETTER-1 trial [10]. Differences exist between the patient cohort here and that included in NETTER-1 which may have influenced the response rate. For example, this cohort was not limited to patients with well-differentiated grade 1 and grade 2 tumors of gastro-entero-pancreatic (GEP) origin. In addition, a limited amount of our patients had not received or shown progression on octreotide which was a requirement of patients in the NETTER-1 trial and may indicate that those patients in our cohort received Lu-177 at an earlier stage of the disease. Imaging may have contributed to the higher rate of PR in this cohort as well. RECIST criteria may underestimate PR by labeling patients as SD due to various reasons when single modality imaging is used [17]. The response assessment of this patient cohort included both morphological and functional imaging with the use of PET/CT in over a third of the patients. This contrasts with the use of only morphologic imaging for the response assessment of NETTER-1 patients and may indicate a more accurate response assessment in the cohort of patients presented here. Overall, it is promising to see that patients in the real world perform well with this therapy. The response rate seen in this group is even more meaningful when considering that no other systemic therapies for patients with GEP-NETs have shown a response rate greater than 5% [18–21].

These findings are promising, however, Lu-177 is not without possibilities of adverse effects including exposure to radiation and myelotoxicity [22–24]. As always, it is vital to determine the population who will most benefit from therapies so that unnecessary toxicities can be avoided. With this motivation, we performed a secondary analysis of various subgroups within our patient cohort. The results of the secondary outcome analysis showed no difference between patients with liver metastasis, with different sites of origin, or with different grades of tumor. However, it did show a significant difference in response rate and disease control rate in patients with non-functional tumors compared to those with functional tumors.

Non-functional NETs are historically associated with later presentation typically related to tumor size and metastasis [25, 26]. Although they produce peptide secretion, the secretions are not at a level to produce the clinical syndrome present in functional tumors [27]. Several studies have shown that patients with non-functional tumors have decreased survival compared to those with functional tumors [28, 29]. One report from Cleveland Clinic showed a 37% difference in 10-year survival between patients with insulinomas and non-functional tumors [28]. Another report from Mayo Clinic showed a 33% difference in survival at 3 years between those with gastrinomas and non-functional tumors [29]. Our finding that Lu-177 has a better response rate in patients with non-functional tumors may prove to be a significant breakthrough for this group of patients who historically have poorer outcomes. To our knowledge, this is a finding that has not been described in the literature previously, and larger randomized studies should be performed to evaluate this finding. In addition, this study is unique in that it includes patients with unknown primary tumors. These patients often represent a challenging group of treatment and further research is needed to assess the utility of PRRT in this patient population. Currently, phase II clinical trials are in the process to evaluate the efficacy and safety of PRRT treatment in combination with immunotherapy and chemotherapy [30, 31]. It is vital that subgroup analyses are performed in these and other future trials to assess for differences among the efficacy of groups such as those with non-functional tumor status or unknown primary tumors.

This study was limited by retrospective chart review and a small patient cohort isolated to a single center site. Further studies should involve multiple centers and prospective comparisons of patient cohorts to validate our findings. In addition, future analysis will need to be performed on this cohort at a later date to obtain PFS and OS data. These analyses could not be performed as a majority of these patients are alive and have not had progression of their malignancy at this time.

In conclusion, our cohort of patients with GEP-NETs treated with Lu-177 not only validated the efficacy of this treatment but showed a superior response rate compared to early trials. This is promising that Lu-177 is a favorable treatment for this patient group with limited options. Furthermore, we found in a subgroup analysis that patients with non-functional tumors had a higher response rate to treatment than those with functional tumors. Further studies should evaluate this finding which could prove to be a significant breakthrough for these patients who historically have poorer outcomes.

## Abbreviations

^68^Ga-PET: gallium-68 dotatate-positron emission tomography
CT: computed tomography
FDA: Food and Drug Administration
GEP-NETs: gastro-entero-pancreatic neuroendocrine tumors
Lu-177: ^177^Lu-dotatate
NETs: neuroendocrine tumors
OS: overall survival
PD: progressive disease
PFS: progression-free survival
PR: partial response
PRRT: peptide receptor radionuclide therapy
RECIST: Response Evaluation Criteria in Solid Tumors
SD: stable disease

## Declarations

### Availability of data and materials

The datasets for this manuscript are not publicly available as it contains patient health-protected information. Requests for accessing the datasets should be directed to the corresponding author Anup Kasi, akasi@kumc.edu.

### Copyright

@ The Author(s) 2023.
